# Acute Respiratory and Cardiovascular Admissions after a Public Smoking Ban in Geneva, Switzerland

**DOI:** 10.1371/journal.pone.0090417

**Published:** 2014-03-05

**Authors:** Jean-Paul Humair, Nicolas Garin, Eric Gerstel, Sebastian Carballo, David Carballo, Pierre-Frédéric Keller, Idris Guessous

**Affiliations:** 1 Division of Primary Care Medicine, University Hospitals of Geneva, Geneva, Switzerland; 2 Division of General Internal Medicine, University Hospitals of Geneva, Geneva, Switzerland; 3 Division of Internal Medicine, Chablais Regional Hospital, Monthey, Switzerland; 4 Division of Ambulatory Care and Emergency Medicine, Clinique La Colline, Geneva, Switzerland; 5 Division of Cardiology, University Hospitals of Geneva, Geneva, Switzerland; 6 Unit of Population Epidemiology, Division of Primary Care Medicine, University Hospitals of Geneva, Geneva, Switzerland; 7 Community Prevention Unit, Lausanne University Hospital, Lausanne, Switzerland; Kyushu University Faculty of Medical Science, Japan

## Abstract

**Background:**

Many countries have introduced legislations for public smoking bans to reduce the harmful effects of exposure to tobacco smoke. Smoking bans cause significant reductions in admissions for acute coronary syndromes but their impact on respiratory diseases is unclear. In Geneva, Switzerland, two popular votes led to a stepwise implementation of a state smoking ban in public places, with a temporary suspension. This study evaluated the effect of this smoking ban on hospitalisations for acute respiratory and cardiovascular diseases.

**Methods:**

This before and after intervention study was conducted at the University Hospitals of Geneva, Switzerland, across 4 periods with different smoking legislations. It included 5,345 patients with a first hospitalisation for acute coronary syndrome, ischemic stroke, acute exacerbation of chronic obstructive pulmonary disease, pneumonia and acute asthma. The main outcomes were the incidence rate ratios (IRR) of admissions for each diagnosis after the final ban compared to the pre-ban period and adjusted for age, gender, season, influenza epidemic and secular trend.

**Results:**

Hospitalisations for acute exacerbation of chronic obstructive pulmonary disease significantly decreased over the 4 periods and were lowest after the final ban (IRR = 0.54 [95%CI: 0.42–0.68]). We observed a trend in reduced admissions for acute coronary syndromes (IRR = 0.90 [95%CI: 0.80–1.00]). Admissions for ischemic stroke, asthma and pneumonia did not significantly change.

**Conclusions:**

A legislative smoking ban was followed by a strong decrease in hospitalisations for acute exacerbation of chronic obstructive pulmonary disease and a trend for reduced admissions for acute coronary syndrome. Smoking bans are likely to be very beneficial for patients with chronic obstructive pulmonary disease.

## Introduction

There is strong evidence that passive exposure to tobacco smoke is harmful to human health [1]. Tobacco smoke has pro-atherogenic and pro-thrombotic effects through various mechanisms [2]. A systematic review of 18 cohort and case-control studies demonstrated that exposure of non-smokers to second-hand smoke (SHS) is associated with a 25% increased risk of coronary artery disease and myocardial infarction [3]. Many studies and meta-analysis showed a moderate, consistent and dose-dependent association between exposure to SHS and the risk of stroke, which is increased by 25%, suggesting a causal relationship [4]–[6]. A strong and consistent association was reported between SHS exposure and several respiratory hazards: respiratory symptoms, worsening of lung function tests, prevalence of chronic obstructive pulmonary disease (COPD) and asthma, risk of COPD or asthma exacerbations and risk of hospital admission for COPD, asthma or pneumonia [7]–[9].

Exposure to SHS causes an important health and economic burden and is a major concern for health policy. Many countries have therefore introduced legislation for smoking bans in public and work places to reduce exposure to tobacco smoke. Numerous studies and 2 meta-analyses have shown that legislation for comprehensive smoking bans are associated with an average 15% reduction of hospital admissions for acute myocardial infarction and coronary events within a year [10]–[15]. Fewer studies and a recent meta-analysis showed that a comprehensive smoke-free legislation is followed by a 19% decrease in hospitalisations for ischemic stroke [10], [14]–[16].

Workplace smoking bans are associated with an improvement of self-reported respiratory symptoms and lung function tests among hospitality employees heavily exposed to SHS [17]–[19]. Hospital admissions for acute respiratory diseases were globally reduced by 24% after implementation of comprehensive smoke-free laws [10], [14]–[16]. The evidence is stronger for asthma among adults and children with a decrease in emergency visits and hospitalisations [8], [10], [14]–[16], [20]. Two studies suggest a significant reduction of admissions for pneumonia and a controversial effect on COPD exacerbations, which does not emerge as significant in the meta-analysis [10], [14], [16].

A legislative smoking ban in public places was implemented after a popular vote on 1 July 2008 in the Canton of Geneva, Switzerland. Three months later, the smoke-free law was cancelled by the Supreme Court, which considered the implementing regulation as unlawful. However many public places remained smoke-free as managers maintained the ban voluntarily. A second vote led to a permanent smoking ban, which was applied from 30 October 2009 [21].

The Department of Health of the Canton of Geneva called for and sponsored this study to evaluate the effect of the public smoking ban on hospital admissions for acute respiratory and cardiovascular diseases at the University Hospitals of Geneva.

## Materials and Methods

### Ethics statement

The Ethics committee of the University Hospitals of Geneva approved the study (protocol 10-241R) on 18 February 2011 and waived the informed consent as only aggregate data from an existing database were used.

### Design and setting

**Figure 1:**
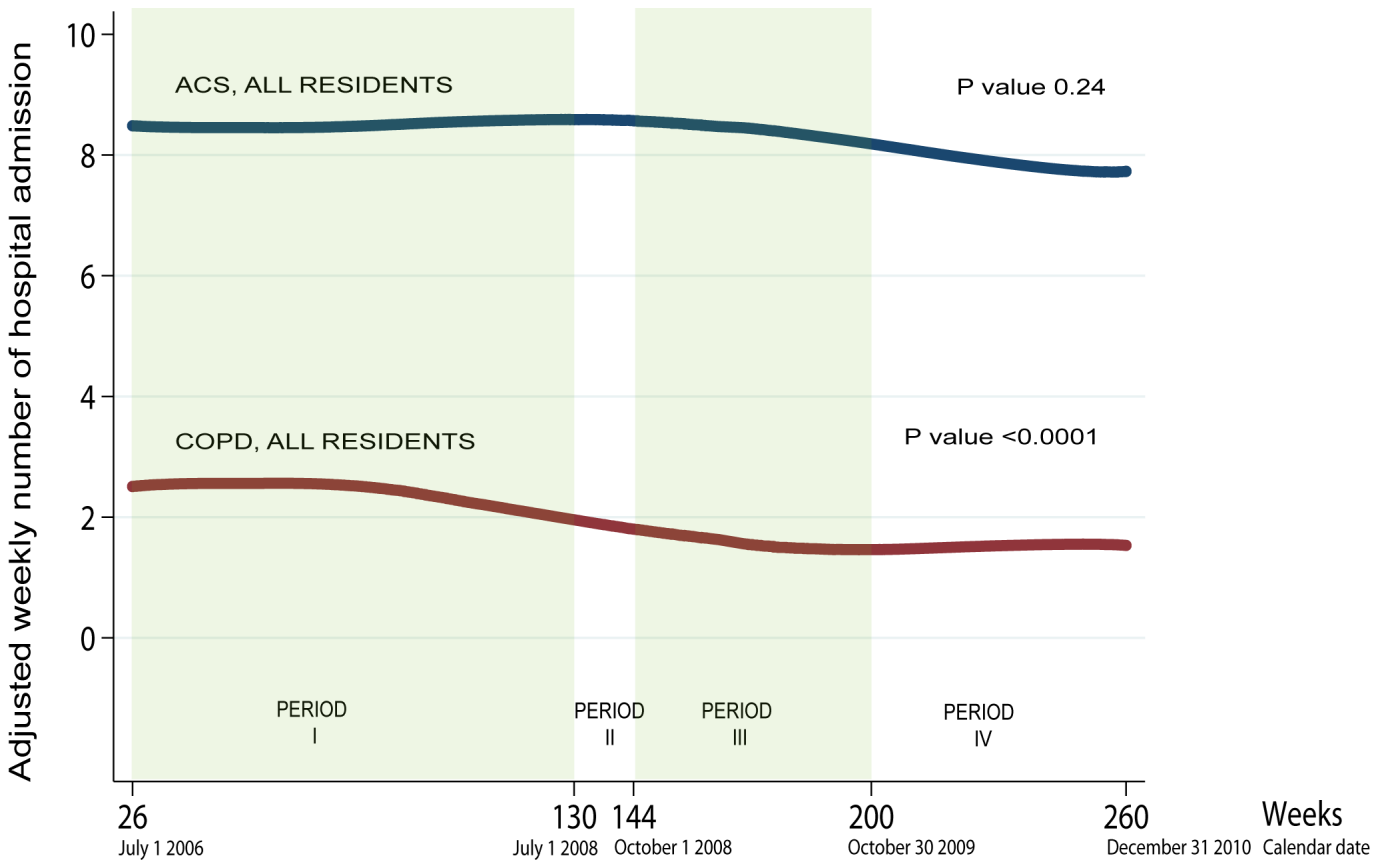
Adjusted weekly numbers of hospitalizations for acute COPD exacerbations and acute coronary syndromes (ACS). Footnote: Global P values for adjusted incidence rate ratio trend tests (reference = period 1, weeks 26 to 130), see Table 2. Curves displayed using smoothed values (Stata *lowess* function).

We conducted this before and after intervention study in the University Hospitals of Geneva, Switzerland, a 1900-bed university and single public hospital in the Canton of Geneva, which is populated by about 450,000 inhabitants. We collected data in four periods defined according to the legislation on a public smoking ban in the Canton of Geneva (Figure 1): the 2-year period before the first smoking ban implemented on 1 July 2008 (Period 1); the 3-month period during the first smoking ban until its suspension by the Supreme Court on 30 September 2008 (Period 2); the 13-month period of suspended smoking ban from 1 October 2008 until the implementation of the final smoking ban on 31 October 2009 (Period 3); and the initial 14-month period of the final smoking ban from 30 October 2009 to 31 December 2010 (Period 4).

### Participants and data collection

From the hospital database of the University Hospitals of Geneva, we extracted data on patients aged 16 and over admitted between 1 July 2006 (104 weeks before the first legislation) and 31 December 2010 (61 weeks after the second legislation) with 5 primary diagnoses identified by the codes of the International Classification of Diseases (ICD-10), 10th Revision [22]: acute coronary syndrome (ICD-10 codes: I 21); cerebrovascular diseases including ischemic stroke and transient ischemic attack (ICD-10 codes: I 63–67, G 45–46); COPD entities among chronic lower respiratory diseases (ICD-10 codes: J 40–44); pneumonia or influenza (ICD-10 codes: J 10–16), and asthma (ICD-10 codes: J 45-46). Like similar studies, we recorded only patients' first hospital stay [12]. Extracted information included date of admission, gender, age, place of residence (Canton of Geneva, other or unknown), and diagnosis at admission.

Because the definition of transient ischemic attack changed in 2009 [23], transient ischemic attack and ischemic stroke diagnoses were combined into a single category (i.e. ischemic cerebrovascular accident). As a quarter of patients included in the study had their residence outside of the Canton of Geneva, which could mitigate the impact of smoke-free legislations in Geneva, we also analysed data restricted to residents of the Canton of Geneva.

### Statistical analyses

The means and frequencies with standard deviation and 95% confidence intervals (95%CI) of study variables were computed. The mean number of weekly admissions for each diagnosis was calculated from the number of diagnosis-specific hospitalisations in each study period. We used Poisson regression models to compute the 95% CIs around these estimates and to test the hypothesis that there was a change in the mean number of weekly admissions for each diagnosis between the study periods corresponding to changes in smoking ban legislation. Dummy variables for the periods were created and the impact of smoking ban legislation was estimated by incidence rate ratios (IRR) of diagnosis-specific hospitalizations for the different periods, considering period 1 as the reference period. Some of the disease-specific hospital admissions, such as acute coronary syndrome, acute exacerbation of COPD, and pneumonia, are known to vary with seasons or to increase during the influenza epidemic [24]. Therefore, regression models were adjusted for season (defined by calendar months) and influenza epidemic periods using surveillance data of the Swiss Office of Public Health [25]–[28]. To account for the potential secular trends of diseases that would be independent of the legislation periods, we included a linear time trend in the models. Models were additionally adjusted for age and gender. The seasonal effect and secular trends were taken into account with methods previously used to allow comparison to previous large population-based studies that measured the impact of smoking bans on health outcomes [12], [15], [16], [20]. We also examined whether the slope of the trend change across periods, by adding an interaction term between the study periods and the linear predictor for time to the models and using likelihood ratio tests [12]. For each outcome, we performed a global trend test of adjusted incidence rate ratio across four periods, using period 1 (weeks 26 to 130) as the reference period, with its result expressed as a global p-value.

To estimate the events prevented, the admission rates obtained among Geneva residents were applied to the counts of population aged over 15, using census data of Geneva for each study period [29]. Hospital days and costs prevented by the smoking ban were estimated using the University Hospitals of Geneva administrative data.

All statistical analyses were performed using Stata 11.0 (Stata Corp, College Station, USA).

## Results

### Patients' characteristics

Overall, 5345 patients with a first hospitalization for acute coronary syndrome, ischemic stroke, acute COPD exacerbation, pneumonia and acute asthma were included (Table 1). Patients were predominantly males, had a mean age of 67 years, stayed on average for 11 days in hospital, and three-quarters of them lived in Geneva. Admissions for acute cardiovascular events were more numerous than for acute respiratory diseases. The socio-demographic profile and admission diagnoses were similar across the 4 study periods and for the sub-sample of Geneva residents.

**Table 1:** Socio-demographic characteristics and diagnoses for hospitalizations for all subjects and Geneva residents.

	Total	Period 1: Before any smoking ban	Period 2: Transient smoking ban	Period 3: Suspended, partial smoking ban	Period 4: Final smoking ban	Geneva residents
***Number of patients***	5345	2411	294	1271	1369	4034
***Study period duration, (weeks)***	234.4	104.3	13	56.3	60.9	234.4
***Gender, N (%)***						
Men	3226 (60.4)	1452 (60.2)	168 (57.1)	764 (60.1)	842 (61.5)	2393 (59.3)
Women	2119 (39.6)	959 (39.8)	126 (42.9)	507 (39.9)	527 (38.5)	1641 (40.7)
***Mean age, years (IC 95%)***	67.1 (66.7–67.5)	67.6 (67.0–68.2)	66.5 (64.7–68.4)	66.6 (65.8–67.5)	66.7 (65.8–67.5)	68.5 (68.0–69.0)
***Place of residence, N (%)***						
Canton of Geneva	4034 (75.4)	1806 (74.9)	199 (67.7)	966 (76.0)	1063 (77.6)	4034
Other	1002 (18.7)	461 (19.1)	70 (23.8)	229 (18.0)	242 (17.7)	NA
Unknown	312 (5.8)	144 (6.0)	25 (8.5)	76 (6.0)	67 (4.9)	NA
***Mean hospital stay, days (IC 95%)***	11.1 (10.8–11.3)	11.1 (10.7–11.4)	11.9 (10.7–13.2)	10.8 (10.3–11.3)	11.1 (10.6–11.7)	11.4 (11.1–11.7)
***Main diagnosis at admission, N (%)***						
Acute coronary syndrome	1973 (36.9)	893 (37.0)	122 (41.5)	484 (38.1)	474 (34.6)	1431 (35.5)
Ischemic cerebrovascular accident	2493 (46.6)	1079 (44.7)	135 (45.9)	610 (48.0)	669 (48.9)	1868 (46.3)
Acute COPD exacerbation	436 (8.2)	253 (10.5)	15 (5.1)	73 (5.7)	95 (6.9)	379 (9.4)
Pneumonia	239 (4.5)	105 (4.4)	9 (3.1)	52 (4.1)	73 (5.3)	195 (4.8)
Acute asthma	204 (3.8)	81 (3.4)	13 (4.4)	52 (4.1)	58 (4.2)	161 (4.0)

NA  =  not applicable.

### Effects of public smoking ban on hospital admissions

The mean number of weekly hospital admissions for all studied conditions differed across periods for all population groups (Table 2). The number of hospitalisations for pneumonia and asthma was particularly low in each study period.

**Table 2:** Impact of smoking ban on hospitalizations for acute respiratory and cardiovascular diseases for all patients and Geneva residents only.

	Period 1: Before any smoking ban	Period 2: Transient smoking ban	Period 3: Suspended, partial smoking ban	Period 4: Final smoking ban	Global P value
	***Number of weekly hospital admissions***				
Acute COPD exacerbation: All patients	2.45 (2.24–2.59)	1.12 (0.84–1.54)	1.33 (1.12–1.47)	1.54 (1.40–1.75)	<0.0001
Acute COPD exacerbation: Geneva residents only	2.17 (1.96–2.31)	0.77 (0.49–1.19)	1.12 (0.98–1.33)	1.33 (1.19–1.54)	<0.0001
Pneumonia: All patients	0.98 (0.91–1.12)	0.70 (0.49–0.98)	0.91 (0.77–1.05)	1.19 (1.05–1.33)*	<0.0001
Pneumonia: Geneva residents only	0.91 (0.77–0.98)	0.35 (0.21–0.70)	0.70 (0.63–0.91)	0.98 (0.84–1.12)	<0.0001
Acute asthma: All patients	0.77 (0.70–0.84)	0.98 (0.77–1.33)	0.91 (0.77–1.05)	0.98 (0.84–1.12)	<0.0001
Acute asthma: Geneva residents only	0.56 (0.49–0.70)	0.70 (0.42–1.05)	0.77 (0.70–0.98)	0.77 (0.70–0.98)*	<0.0001
Acute coronary syndrome: All patients	8.54 (8.26–8.89)	9.38 (8.47–10.36)	8.61 (8.19–9.03)	7.77 (7.42–8.19)	<0.0001
Acute coronary syndrome: Geneva residents only	6.16 (5.88–6.51)	6.79 (5.88–7.77)	6.23 (5.81–6.65)	3.28 (5.39–6.16)	<0.0001
Ischemic stroke: All patients	10.36 (10.01–10.71)	10.36 (9.45–11.41)	10.85 (10.36–11.34)	10.99 (10.57–11.48)	<0.0001
Ischemic stroke: Geneva residents only	7.56 (7.28–7.91)	6.72 (5.81–7.70)	8.83 (7.84–8.82)	8.61 (8.12–9.03)	<0.0001
	Adjusted incidence rate ratios**				
Acute COPD exacerbation: All patients	ref	0.42 (0.25–0.71)*	0.42 (0.33–0.54)*	0.54 (0.42–0.68)*	<0.0001
Acute COPD exacerbation: Geneva residents only	ref	0.36 (0.19–0.68)*	0.42 (0.32–0.55)*	0.53 (0.41–0.68)*	<0.0001
Pneumonia: All patients	ref	0.58 (0.30–1.15)	0.74 (0.54–1.02)	1.00 (0.75–1.35)	0.12
Pneumonia: Geneva residents only	ref	0.43 (0.18–1.06)	0.71 (0.49–1.01)	0.97 (0.70–1.35)	0.08
Acute asthma: All patients	ref	1.10 (0.61–1.97)	1.14 (0.81–1.60)	1.17 (0.82–1.66)	0.81
Acute asthma: Geneva residents only	ref	1.06 (0.53–2.15)	1.28 (0.87–1.87)	1.36 (0.91–2.01)	0.42
Acute coronary syndrome: All patients	ref	1.06 (0.88–1.29)	0.98 (0.88–1.09)	0.90 (0.80–1.00)	0.24
Acute coronary syndrome: Geneva residents only	ref	1.18 (0.95–1.48)	0.98 (0.86–1.12)	0.89 (0.78–1.02)	0.11
Ischemic stroke: All patients	ref	0.92 (0.77–1.11)	0.95 (0.86–1.04)	0.98 (0.89–1.08)	0.64
Ischemic stroke: Geneva residents only	ref	0.89 (0.71–1.11)	0.96 (0.86–1.07)	0.99 (0.89–1.11)	0.67

* P<0.05 compared to period 1; ** adjusted for age, gender, season, influenza epidemic and secular trend; ref  =  reference period.

For acute COPD exacerbation, the weekly number of hospitalisations significantly dropped over the 4 periods from 2.45 to 1.54 (p<0.0001) (Table 2). The adjusted IRR decreased significantly in all periods after the initial smoking ban; it reached 0.54 [95%CI: 0.42–0.68] for all patients and 0.53 [95%CI: 0.41–0.68] for Geneva residents during the final smoking ban (period 4). There was evidence of a change in the slope of the trend line across the study periods (P = 0.001 for periods x secular trends interaction). The reduction started before the legislative smoking ban and became stable in the second part of the period 3, when the ban was legislatively suspended but partially maintained in practice (Figure 1).

Hospitalizations for acute coronary syndrome decreased only during the final smoking ban (period 4), where we measured a trend close to significance: adjusted IRR was 0.90 [95%CI: 0.80–1.00] for all patients and 0.89 [95%CI: 0.78–1.02] for Geneva residents (Table 2). The slope of the trend line did not change across the periods (P>0.05 for periods x secular trends interaction). The reduction started after the first legislative smoking ban was suspended but partially maintained (period 3) (Figure 1).

Despite variations in the number of admissions, adjusted IRR of hospitalisations for pneumonia, acute asthma and ischemic stroke, did not significantly change throughout the 4 periods.

## Discussion

This study shows that a legislative smoking ban is associated with a strong, highly significant reduction in hospitalisations for COPD exacerbation and a trend for decreased admissions for acute coronary syndrome. We observed no significant change in admissions for pneumonia, acute asthma and ischemic stroke.

### Strengths and limitations

Among the strengths of this study is the large sample covering the large majority of hospital admissions in the community. It investigated the impact of a smoking ban on several types of acute respiratory and cardiovascular events, including some that were less explored. In addition, it used a robust Poisson regression analysis to better assess the specific effect of the smoking ban and minimize confounding effects, particularly secular trends and interaction with influenza epidemics.

The use of data from a single hospital is a study limitation but the findings are probably generalizable for the Canton of Geneva. Indeed, hospitalisations for acute events in the Canton of Geneva are mainly centralized to the single acute care hospital of the University Hospitals of Geneva, which is the only public hospital of the Canton. For example, more than 90% of patients with an acute coronary syndrome are admitted in this institution. As we analysed retrospectively administrative hospital data, inaccurate coding of ICD-10 diagnoses at admission is possible and could potentially lead to misclassification bias. This might particularly affect patients admitted for combined acute COPD exacerbation and pneumonia, leading eventually to overestimate the decrease in admissions for COPD exacerbations. The hospital database only includes data on patients who were admitted to a ward and therefore patients who had ambulatory treatment were not considered. This latter limitation particularly affects data on patients admitted for acute asthma and pneumonia, who are mainly managed as outpatients. Moreover, as we do not have individual data on tobacco use and exposure to SHS, we were unable to determine if the observed decrease in COPD exacerbations was due to a decrease in cigarette consumption among active smokers or to a reduction in exposure to SHS, or both. Finally we did not conduct further stratified analyses (e.g. by age group) because of the limited number of events.

### Effects of public smoking ban on hospital admissions for acute respiratory diseases

This study is the first to show such a large decrease in admissions for acute COPD exacerbation after implementation of a smoking ban in public places. Although it was little explored, this effect was larger than expected, quickly detectable and progressive. A COPD exacerbation is a complex event involving bacterial or viral infections, constitutional factors, active smoking, exposure to SHS and other environmental pollutants, which synergistically induce a flare of inflammation in the lower airways [30]. The observed decrease in hospital admissions may have been due to reduced exposure to SHS and/or a decrease in active smoking induced by the ban [11] but the distinction was not possible in our study. In a Californian cohort, the risk of emergency department visit was 40% higher among COPD patients exposed to SHS than in non-exposed patients [31]. Exposure to SHS was an independent risk factor for readmission in a cohort of Spanish patients recruited during a hospitalization for COPD exacerbation [7]. The 46% reduction of admissions observed in our study was larger than the 27% decrease reported after a comprehensive stepwise smoking ban in Toronto [14] and the absence of change after implementation of the work place smoking ban in Ireland [16]. These differences might be partly explained by the different methods of adjustment between studies: the Canadian study adjusted only for secular trend while the Irish study also used a Poisson regression but adjusted for the seasonal temperature, levels of particulate matter PM 2.5 and PM 10 and the intensity of influenza epidemics. Finally, a secular trend is unlikely to explain this decrease as the number of hospitalisations for COPD in Switzerland declined by 27% from 2000 to 2006, but stabilized thereafter. In our study, the reduction in hospitalisations was larger (46%) and occurred later (2006–2010) [32].

Admissions for COPD exacerbations decreased progressively before the first smoking ban. This effect can be explained by the progressive introduction of smoke-free work and public places, from 2003, before our first 2-year study period preceding the first legislative smoking ban. Since 2003, the local tobacco control association has offered support to companies, public services and associations to implement smoke-free workplaces. Most of this activity took place before study period 1, between October 2003 and June 2006 and reached 93 groups and more than 65′000 persons. The activity decreased during the study period 1, when it reached 35 groups and about 6500 persons (Unpublished data).

To estimate the public health impact from our results, the smoking ban could prevent yearly 47 new hospitalisations for acute COPD exacerbations, 680 hospital days and costs of CHF 1.28 millions (US$ 1.38 millions). As COPD exacerbations are associated with deterioration of lung function, increased mortality and decreased quality of life, a smoking ban has the potential to bring considerable gains both for patients' health and the public health system, with a magnitude similar to therapeutic interventions.

We did not find a significant reduction in hospitalisations for asthma or pneumonia, as reported in other studies [8]–[10], [14], [16], [20]. Instead, we observed a slight, progressive but non-significant trend towards an increase in admissions for acute asthma throughout the periods. Although there is no clear interpretation for this finding, it may be partly explained by the very few cases of admissions for acute asthma and pneumonia during the short summertime period corresponding to the initial smoking ban. The 2009 influenza pandemic had its major impact at a time that overlaps the end of the suspended ban and the beginning of the final smoking ban. As we used the same adjustment for influenza pandemic and seasonal epidemics, the impact of the influenza pandemic on admissions for acute asthma and pneumonia was probably underestimated in our model. There is evidence showing that a third of patients hospitalized during the 2009 influenza pandemic had underlying respiratory disease and that admission rates of patients aged under 65 were 6 times higher than during seasonal epidemics [33], [34]. Finally, as we did not include patients with ambulatory treatment of pneumonia and acute asthma, our sample was possibly too small to detect an effect of the smoking ban on these outcomes.

### Effects of public smoking ban on hospital admissions for acute cardiovascular diseases

This study shows that the smoking ban was followed by a trend towards a 10% decrease in hospitalisations for acute coronary syndrome. Although the effect is not significant and lower than in the largest meta-analysis (−15%), it is within the range of previous results [10]. For example, an English study observed an even smaller reduction of admissions for myocardial infarction (−2.4%), which was significant in a very large national sample [12]. Like in England, this lower effect can be due to the progressive reduction of exposure to SHS because of stepwise implementation of smoking restrictions before the first legislative smoking ban, as described above. It is likely that a longer study with a larger sample would show a significant reduction in hospitalizations for acute coronary syndrome in Geneva. A seasonal fluctuation might cause the moderate and non-significant reduction of incidence of acute coronary syndrome. This is however unlikely as the incidence would be expected to increase in autumn and winter after the implementation of the final ban [35].

We did not find any association between the smoking ban and admissions for ischemic stroke. However our finding is within the same range as other individual studies, which did not report a significant effect [10], [14], [16]. As there is evidence of an impact of smoke-free legislation on ischemic stroke but not on transient ischemic attack, a hypothetical effect may have been masked by the combination of these diagnoses into the same category of stroke.

### Conclusions

The implementation of a legislative smoking ban is associated with a strong reduction in hospitalisations for acute exacerbations of COPD. This effect is even greater than the known reduction of admissions for acute coronary syndrome. If confirmed in other research, legislative smoking bans are likely to be beneficial for both individual patients and as well as for the health care system as it may be a very effective intervention to reduce COPD morbidity.
